# Genitourinary and Syphilitic Diseases

**Published:** 1903-11

**Authors:** Byron H. Daggett

**Affiliations:** Buffalo, N. Y.


### Genitourinary and Syphilitic Diseases.

CONDUCTED BY BYRON H. DAGGETT, M. D., Buffalo, N. Y.

### THE SURGERY OF THE PROSTATE FROM THE STANDPOINT OF PERSONAL EXPERIENCE.

GRANVILLE MACGOWAN (*New York Medical Journal,* June 13, 1903), discusses the radical surgery for relief of urinary obstruction by encroachment of the chronically inflamed prostate. He does not believe that "once a prostatic always a prostatic."

Speaking from personal experience in nearly a hundred cases, the author states no man who is prostatic, unless the disease be cancerous, need feel that he has a disease of which there is more risk to his life by surgery than otherwise. Many cases can be entirely cured. The tonicity of the bladder may be restored by removal of the obstruction. The kidneys functionate normally after the removal of the obstruction. The lagging mind, the dragging step, the rebellious stomach, the pallid cheek, and the sexual power are restored by surgical relief of urinary obstruction.

Belfield, of Chicago, was probably the first to deliberately plan and execute prostatectomy. Prostatectomy is a shelling out or excochleation of the gland either through a perineal or suprapubic opening. Posture of the patient is more important and of greater assistance than any of the devices suggested for traction. The posture referred to is exaggerated lithotomic and the abstractor would still improve this position by raising the body from the hips up, to an angle of 20 degrees from the horizontal line.

Sometimes these tumors are so dense and adherent that they must be removed piecemeal with rongeurs or serrated scissors, guided by the index finger. This procedure is very tedious and bloody sometimes, yet very satisfactory in results.

When the obstruction to urination becomes so great as to demand frequent or regular catheterisation, surgical interference is in order. The catheter is a constant menace to life, for in spite of all warnings and care, urinary infection comes about in the majority of cases, a condition worse than any hazard of operation. No one is too old for these operations. Successful Bottini operations and successful prostatectomies have been done upon men who have passed the age of 80.

Albumin and casts in the urine, stone in the bladder and weakness are not contraindications to operation. Pus, blood, albumin in limited quantities, extreme age, sickness, feebleness, within reasonable limits or septic symptoms do not prohibit operation.

There are three contraindications to operation: (1) a tendency to bleed from very slight injuries; (2) serious heart lesion, accompanied with great muscular weakness; (3) and most important, is urinary insufficiency.

Even in a deficiency of kidney action, a bad pyelitis, or great feebleness, a Bottini operation may be done for immediate relief, followed subsequently by a prostatectomy. A scar left after galvano cautery does not seriously interfere with prostatectomy. If the structure be cancerous the Bottini is the operation of choice, and carried out through a perineal section.

Some authors teach that all prostatectomies should be done through the suprapubic route, others through the perineal. If the tumors be fibroid or myomatous and located high, they can not be removed through the perineal opening; they can not be peeled out; they can only be taken piecemeal by rongeur. Adenoid nodules will peel. If the outer capsule of the prostate or the neck of the bladder is not torn, the hemorrhage will not be serious. Sclerotic arteries or rupture of enlarged veins may lead to severe hemorrhage, which may be controlled by the use of water at 120° F. The perineum, scrotum and buttocks should be protected by vaseline, or the wound packed with gauze, moistened with a solution of 1 to 1000 of adrenalin chloride. The gauze should be carried into the capsule. Give two quarts of normal salt solution by enema every three hours until secretions come free.

---

### CYSTITIS IN THE FEMALE.

CHARLES D. LOCKWOOD (*Southern California Practitioner*), April, 1903,) states that four distinct types of vesical inflammation have come under his observation in gynecological practice:

(1) irritable bladder—this form occurs in young, unmarried and fat, sluggish women. Symptoms are frequent smarting and burning micturition. Urine normal, no pus, no blood. The cystoscope shows hyperemia of the trygone. The urethra is congested and hyperesthetic. Urine often excessively acid. These cases must be differentiated from true cystitis by chemical and microscopical examination and inspection of the bladder. The second group of cases are due to infection by pus forming microbes, such as the colon bacillus, proteus albus, staphylococcus and streptococcus. The infection reaches the bladder by the urine, by unclean instrumentation and by penetration of the tissues from neighboring inflammation and abscess. Retention and decomposition of urine are the most common predisposing factors in causing this malady. Retention is often due to cystocele. This group is the most amenable to treatment and the most common. Diagnosis is made by physical, chemical and cystoscopic examinations. The third group is due to tubercular lesions; the fourth variety is due to gonorrhea.

